# Immunological effects of the intraparenchymal administration of allogeneic and autologous adipose-derived mesenchymal stem cells after the acute phase of middle cerebral artery occlusion in rats

**DOI:** 10.1186/s12967-018-1709-y

**Published:** 2018-12-05

**Authors:** Zhang Yu, Tang Wenyan, Su Xuewen, Dong Baixiang, Wang Qian, Wang Zhaoyan, Yang Yinxiang, Qu Suqing, Luan Zuo

**Affiliations:** 1 Department of Pediatrics, Navy General Hospital, No. 6, Fucheng Road, Haidian District, Beijing, 100048 China; 2 Department of Neonatal Intensive Care Unit, Beijing Obstetrics and Gynecology Hospital, Capital Medical University, No. 251, Yaojiayuan Road, Chaoyang District, Beijing, 100026 China; 3 Beijing Yinfeng Dingcheng Bioengineering Technology Co., Ltd., No. 14, Zhonghe Street, Yizhuang Economic and Technological Development Zone, Daxing District, Beijing, 100176 China

**Keywords:** Autologous, Allogeneic, Adipose tissue-derived stem cells, Transplantation, Focal cerebral infarct

## Abstract

**Background:**

Adipose-derived mesenchymal stem cell (ADMSC) therapy can promote recovery from cerebral ischemia; however, more information regarding appropriate sources of ADMSCs is required. This study was aimed at analyzing the immunogenicity of rat ADMSCs by comparing the immunological effects of intraparenchymal administration of allogeneic ADMSCs (allo-ADMSCs) and autologous ADMSCs (auto-ADMSCs) after the acute phase of middle cerebral artery occlusion (MCAO) in rats.

**Methods:**

Allo- or auto-ADMSCs from rats (1 × 10^6^ cells) were transplanted into Lewis rats 8 days post MCAO. The immunogenicity of ADMSCs was analyzed using coculture with T lymphocytes. The in vivo immune response induced by rat ADMSCs and the viability, migration, and differentiation of transplanted ADMSCs were detected using immunohistochemistry. Apoptosis within the populations of transplanted cells were detected using a TUNEL assay. Infarct volume was detected by 2,3,5-triphenyltetrazolium chloride staining. Post-treatment neurological function was evaluated using a modified neurological severity score and rotarod test. Data were analyzed using Kruskal–Wallis and Mann–Whitney U tests.

**Results:**

Compared with allo-ADMSCs, auto-ADMSCs showed lower immunogenicity and evoked weaker immunological responses. Allo-ADMSCs evoked significantly stronger protein expression of interleukin-2 and interferon-gamma, as well as the local accumulation of CD4+ T lymphocytes, CD8+ T lymphocytes, and microglial cells. This indicates that auto-ADMSCs may contribute to higher survival rates, longer survival time, wider migratory scope, and fewer apoptotic cells. In addition, a small number of transplanted auto-ADMSCs expressed astrocyte-like and neuron-like markers 28 days after transplantation. We did not observe surviving transplanted allo-ADMSCs at this time point. We also found that auto-ADMSCs induced a greater degree of functional recovery and a greater reduction in infarct volume than allo-ADMSCs 28 days after transplantation.

**Conclusions:**

Auto-ADMSCs were more effective than allo-ADMSCs in promoting recovery and reducing the infarct volume of MCAO rats. This could be associated with better viability, migratory ability, and differentiation potential, as well as a lower rate of apoptosis. Confirmation of the superiority of auto-ADMSCs and clarification of the underlying mechanisms will provide a theoretical basis for the improved clinical treatment of cerebral infarction.

## Background

Cerebral infarct or ischemic stroke is widely regarded as a major cause of disability and mortality [1]. No ideal treatment of cerebral infarct exists, and clinical outcomes are often poor. Many patients are left with permanent disabilities, including hemiplegic aphasia [2, 3]. Cell-based therapy is an interesting and emerging area of treatment, and is being explored for the treatment of strokes using embryonic stem cells [4], marrow-derived mesenchymal stem cells (BMSCs) [5, 6], and adipose-derived mesenchymal stem cells (ADMSCs) [7].

Compared with embryonic stems cells and BMSCs, ADMSCs are advantageous because they are abundant, easily obtainable with minimal invasiveness, and readily culturable to a sufficient number for autologous transplantation without raising ethical concerns. Furthermore, ADMSCs have a shorter proliferation cycle than BMSCs and produce higher amounts of vascular endothelial cell growth factor and hepatocyte growth factor. A previous study demonstrated the therapeutic superiority of ADMSCs over BMSCs in an animal model of ischemic stroke, and particularly in improving brain function and reducing infarct size [8]. In addition, a recent in vitro study showed that human allogeneic ADMSCs (allo-ADMSCs) have a lower immunogenicity than allogeneic BMSCs and evoke a weaker induction of cytotoxic T-lymphocytes [9]. However, a direct comparison of the immune responses elicited by and therapeutic effects of autologous (auto-) versus allo-ADMSCs has not been made to date.

Therefore, in this study, we aimed to clarify the superiority of auto-ADMSC transplantation and to analyze the underlying mechanisms in rats according to the performance of auto-ADMSCs in terms of the induction of the immune response, migration, survival, apoptosis, and differentiation; the improvement of neurological function; and the reduction of infarct size. Ultimately, these analyses were aimed at providing a theoretical basis for the establishment of a more effective method for treating patients with cerebral infarction.

## Methods

### Experimental groups

Ninety pathogen-free adult male Lewis rats and 10 adult male Brown Norway (BN) rats (Beijing Vital River Laboratory Animal Technology Co., animal license number SCXK, Beijing 2012-0001) with a body weight range of 260–280 g were housed at 23 ± 2 °C, under a relative humidity of 45 ± 15%, and a light/dark cycle of 12 h (lights on: 07:00 to 19:00), with free access to food and water. All Lewis rats underwent surgery to remove adipose tissue (see below) for the preparation of auto-ADMSCs and to induce middle cerebral artery occlusion (MCAO). All BN rats underwent surgery to remove adipose tissue (see below) for preparing allo-ADMSCs; MCAO was not induced. After establishing MCAO, the Lewis rats were randomly assigned to three experimental groups with 30 animals in each study group: an auto-ADMSC group, receiving autologous ADMSC transplantation; an allo-ADMSC group, receiving allogeneic ADMSC transplantation; and a control group receiving only sterile saline. All experiments were designed to minimize animal suffering in compliance with Beijing’s Navy General Hospital’s Ethical Committee guidelines for the Care and Use of Animals in Research. The researchers responsible for functional evaluation and molecular and histological studies were blinded to the treatment groups.

### Isolation of ADMSCs from rats

All 90 Lewis rats and 10 BN rats were anesthetized via an intraperitoneal injection of 6% chloral hydrate at 6 mL/kg body weight. Inguinal fat pads were carefully excised under sterile conditions, cut into < 1-mm^3^ pieces, and incubated with 0.125% type I collagenase solution (Sigma-Aldrich, St. Louis, MO, USA) at 37 °C for 80 min under constant agitation. After three washes with sterile saline, cell suspensions were prepared by gentle homogenization through a 100-µm cell strainer. Cells were collected via centrifugation, and a red blood cell lysing reagent was subsequently added (three times the volume of cells) (Noble Ryder Technology Co. Ltd., Beijing, China, Beijing, China). After pyrolysis for 5 min at room temperature, two sterilized saline washes were performed. Then, the cells were cultured in a serum-free medium (Sigma-Aldrich) for ADMSCs. Third-passage ADMSCs were used in subsequent experiments.

Cells were incubated with the appropriate antibodies in the dark at 4 °C for 30 min to confirm the presence or absence of MSC surface markers and major histocompatibility complex (MHC) classes I and II by flow cytometry before transplantation. Antibodies used to detect MSC surface markers were PE-conjugated anti-rat CD29 (Biolegend, Santiago, CA, USA) and CD90 (Biolegend). Antibodies used to detect MHC class I and II were the anti-MHC class I RT Ia antibody [OX-18] (Biolegend) and the anti-MHC class II antibody [MRC OX-6] (Biolegend). The antibody PE-IgG1 (Biolegend) was used as an isotype control.

### Coculture of ADMSCs and spleen lymphocytes in vitro

ADMSC immunogenicity was analyzed using co-culture with T lymphocytes. T lymphocytes were extracted and purified from the spleens of the Lewis rats and were cocultured for 3 days with auto- or allo-ADMSCs at a proportion of 1:10 in serum-free medium for ADMSCs. Then, the expression levels of IL-2 (eBioscience, San Diego, CA, USA) and IFN-γ (R&D Systems, Minneapolis, MN, USA) were detected using ELISA kits according to the manufacturers’ instructions.

### ADMSC labeling before autologous transplantation

In order to track the transplanted ADMSCs in vivo, third-passage ADMSCs were plated at a density of 1 × 10^6^ cells per flask (T25) and then transduced with lentivirus expressing enhanced green fluorescence protein (EGFP) (Shanghai Genechem Co. Ltd., Shanghai, China) 3 days prior to transplantation. The optimal multiplication of infection (MOI) in transduction medium containing 8 mg/L polybrene was 20. The medium was replaced 24 h after transduction. The efficiency of transduction was assessed by monitoring *EGFP* gene expression by fluorescence microscopy. Cell viability was determined prior to transplantation by trypan blue staining. Labeled cells were used for transplantation at a concentration of 1 × 10^5^ cells/µL.

### Animal model of acute ischemic stroke

After ADMSCs were cultured for 2–3 weeks, acute stroke was induced in the Lewis rats that had undergone adipose-removal surgery. A transverse neck incision was made to expose the right common carotid artery, where a small incision was made to permit the insertion of a 0.28-mm diameter nylon filament. The filament was advanced into the distal right internal carotid artery to occlude the right middle cerebral artery, inducing brain infarction at the blood supply region. Two hours after occlusion, the nylon filament was removed, followed by closure of the muscle and skin layers. Neural function was evaluated using the Zea Longa 5-grade scale [10]. Seventy-five animals with a score of 1–3 points were used in subsequent transplantation experiments.

### Transplantation of ADMSCs

Eight days after the induction of ischemia, the animals were anesthetized and fixed to a stereotactic apparatus. The skull was exposed, and a burr hole was made 3 mm posterior and 1.9 mm lateral from the bregma using a small dental drill. A microinjector was inserted 2.9 mm into the brain parenchyma from the surface of the dura mater, and 10 µL of cell suspension (approximately 1 × 10^6^ ADMSCs) or an equal volume of physiological saline was injected into the brain parenchyma over a period of 20 min.

### Apoptosis of transplanted ADMSCs

Apoptosis within populations of transplanted cells was detected using the TdT-mediated dUTP nick-end labeling (TUNEL) assay. One day after transplantation, the apoptosis of transplanted ADMSCs was detected in the peri-infarct zone using a TUNEL assay kit according to the manufacturer’s instructions (Beyotime, Shanghai, China). Cells were counted in one brain tissue section of each animal (n = 5 per group). The number of double-staining-positive (red and green fluorescence) cells was counted in a minimum of 10 microscopic fields based on their nuclear morphology, and dark color was quantified using a 40× objective and Image-Pro image analysis software.

### Viability, migration, differentiation, and immunological effects

The viability, migration, and differentiation of transplanted ADMSCs, and the in vivo local accumulation of CD4+ T lymphocytes, CD8+ T lymphocytes, and microglial cells induced by rat ADMSCs, were detected using immunohistochemistry. The determination of concentrations of IL-2 and IFN-γ in the brain induced by transplanted ADMSCs was determined using an ELISA assay.

Five rats from each group were anesthetized at 1, 7, and 28 days after transplantation. After perfusion with normal saline and 4% paraformaldehyde and dehydration with 15 and 30% glucose, brain tissues were harvested to produce cryosections. The localization of transplanted cells was observed and photographed under an inverted fluorescence microscope (Olympus IX51, Olympus Corporation, Tokyo, Japan). After EGFP markers were assessed quantitatively with the image analysis software Image-Pro Plus 6.0 (Media Cybernetics, Rockville, MD, USA), the survival rate of the transplanted cells in each group was calculated.

To detect the differentiation potential of transplanted ADMSCs at 8 days post infarction, twenty 10-µm-thick cryosections of the infarcted tissue from each group (n = 5 per group) were examined using immunofluorescent antibodies to mark astrocytes with glial fibrillary acid protein (GFAP; monoclonal antibody diluted 1:1000, incubation at 4 °C overnight, Abcam) to investigate lymphocyte infiltration and microglial proliferation and activation. At 28 days post infarction, additional cryosections were similarly examined using neuronal nuclear antigens (NeuN; monoclonal antibody diluted 1:300, at 4 °C overnight, Abcam) to analyze the differentiation of transplanted ADMSCs.

The distribution of CD4+ and CD8+ T lymphocytes and CD68+ and Iba-1+ microglias around transplanted ADMSCs was also investigated using immunohistochemistry. To this end, these cells were labeled using anti-CD4 antibody (1:200 dilution, incubation at 4 °C overnight, Abcam), anti-CD8 antibody (1:200 dilution, incubation at 4 °C overnight, AbD Serotec, Kidlington, UK), anti-CD68 antibody (1:100 dilution, incubation at 4 °C overnight, Abcam), and the anti-Iba1 antibody (1:100 dilution, incubation at 4 °C overnight, Abcam), respectively. Red fluorescence-labeled (Alexa Fluor 594) secondary antibodies (Jackson ImmunoResearch, Hamburg, Germany) were diluted to 1:100 prior to use. Double-staining-positive (red and green fluorescence) cells were counted as previously described.

To determine the concentrations of IL-2 and IFN-γ in the brain as induced by transplanted ADMSCs, ELISA assays were performed 7 days after transplantation. Five rats from each group were euthanized. Brain tissues from the hippocampus of the graft side were separated and ground on ice and then centrifuged at 5000×*g* for 5 min at 4 °C. Total protein in the supernatant was quantified using a BCA assay. Concentrations of IL-2 and IFN-γ were then determined using ELISA kits according to the manufacturer’s instructions. The protein expression levels of these factors in the brain tissue were expressed as nanogram per milligram total protein.

### Assessment of motor function

Motor function was assessed at 14 and 28 days post transplantation using the modified Neurological Severity Score (mNSS) and rotarod test by two investigators blinded to the experimental groups. The mNSS test is a composite of motor, sensory, balance, and reflex tests. Neurological function was graded on a scale of 0–18, where 0 is normal and 18 represents maximum deficit. In the rotarod test, the 10-cm diameter rotarod cylinder was accelerated from 5 to 40 rpm over 5 min. The time remaining on the rotarod was measured at 0, 14, and 28 days post transplantation, and the data were presented as the mean duration from three trials.

### Assessment of relative cerebral infarction volume

In order to assess the relative cerebral infarction volume at 28 days post transplantation, 2,3,5-triphenyltetrazolium chloride (TTC; Sigma-Aldrich) staining was performed. Five animals from each group were anesthetized using 6% chloral hydrate. After the left ventricle was perfused with 200 mL physiological saline, the brain was removed, sliced into 2-mm sections, stained with 2% TTC for 30 min at 37 °C in the dark, and then fixed with 4% paraformaldehyde. Under these conditions, live tissues retain a red color, while infarcted areas appear white. The ischemic area of the brain was quantified using ImageJ software (NIH, Bethesda, MA, USA), and ischemic volume was expressed as the percentage relative to the ipsilateral hemisphere.

### Statistical analysis

Statistical analysis was performed using SPSS 17 for Windows (IBM, Chicago, IL, USA). Quantitative data are shown as mean values ± SDs. The Kruskal–Wallis test followed by the Mann–Whitney U test were used to compare functional evaluation scores, survival rates of transplanted ADMSCs, numbers of apoptotic transplanted ADMSCs, cerebral infarction volumes, and the expression of IFN-γ and IL-2 in each group. Values of *p* < 0.05 were considered significant at 95% confidence.

## Results

### Characterization of ADMSCs from Lewis and BN rats

**Fig. 1:**
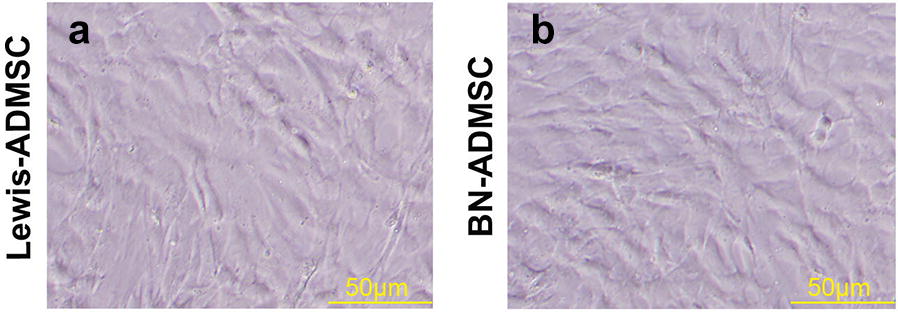
Micrographs of the morphology of ADMSCs from Lewis (**a**) and BN (**b**) rats (×400)

**Fig. 2:**
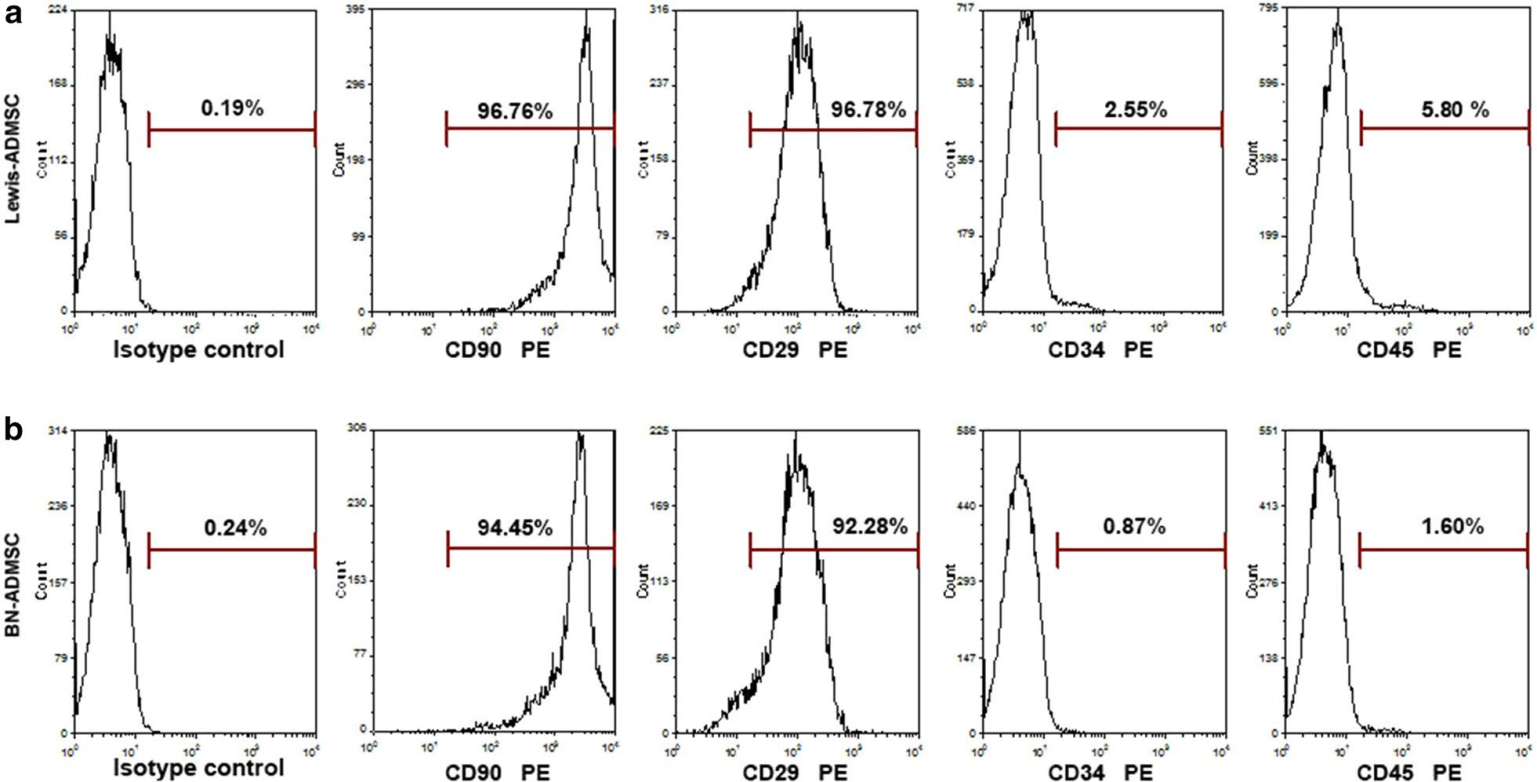
Expression of mesenchymal (CD90, CD29) and non-mesenchymal (CD34, CD45) stem-cell surface markers in transplanted cells from Lewis (**a**) and BN (**b**) rats as measured by flow cytometry

**Fig. 3:**
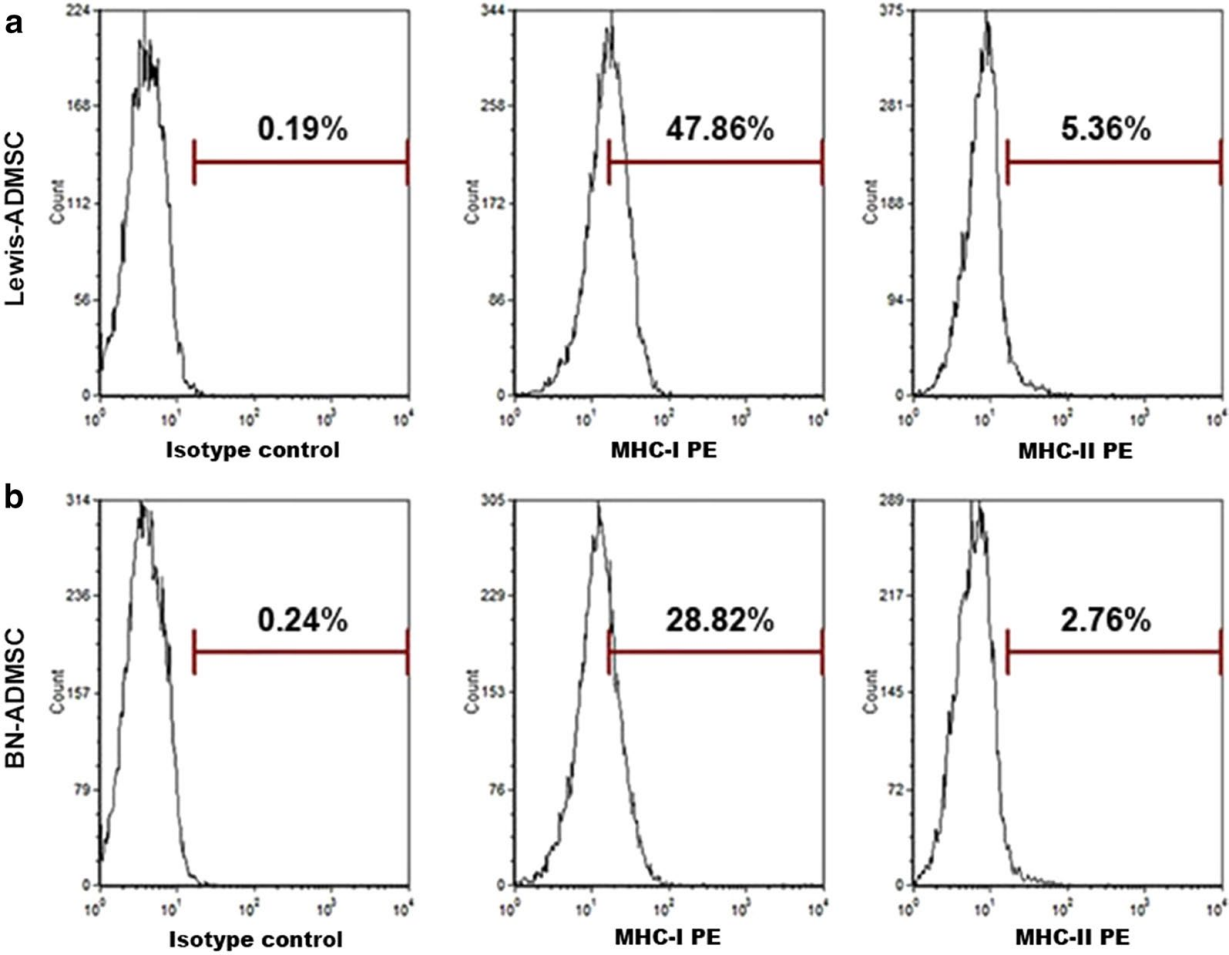
Expression of MHC class I and II markers in transplanted cells from Lewis (**a**) and BN (**b**) rats as measured by flow cytometry

The third-passage cultured ADMSCs waiting for transplantation showed typical, fibroblast-like cell morphology (Fig. 1). Cultured cells from Lewis (96.78%) and BN (92.28%) rats were positive for the CD90-PE surface marker. Cultured cells from Lewis (96.76%) and BN (94.45%) rats were also positive for the CD29-PE surface marker. Non-MSC surface markers (CD34 and CD45) were expressed in less than 6% of cultured cells from Lewis and BN rats (Fig. 2). The MHC class I marker was expressed in 47.86% of the cultured cells from Lewis rats and in 28.82% of the cultured cells from BN rats, while the MHC class II marker was expressed in 5.36% of the cultured cells from Lewis rats and 2.76% of the cultured cells from BN rats (Fig. 3).

### Immunogenicity of ADMSCs in vitro

**Fig. 4:**
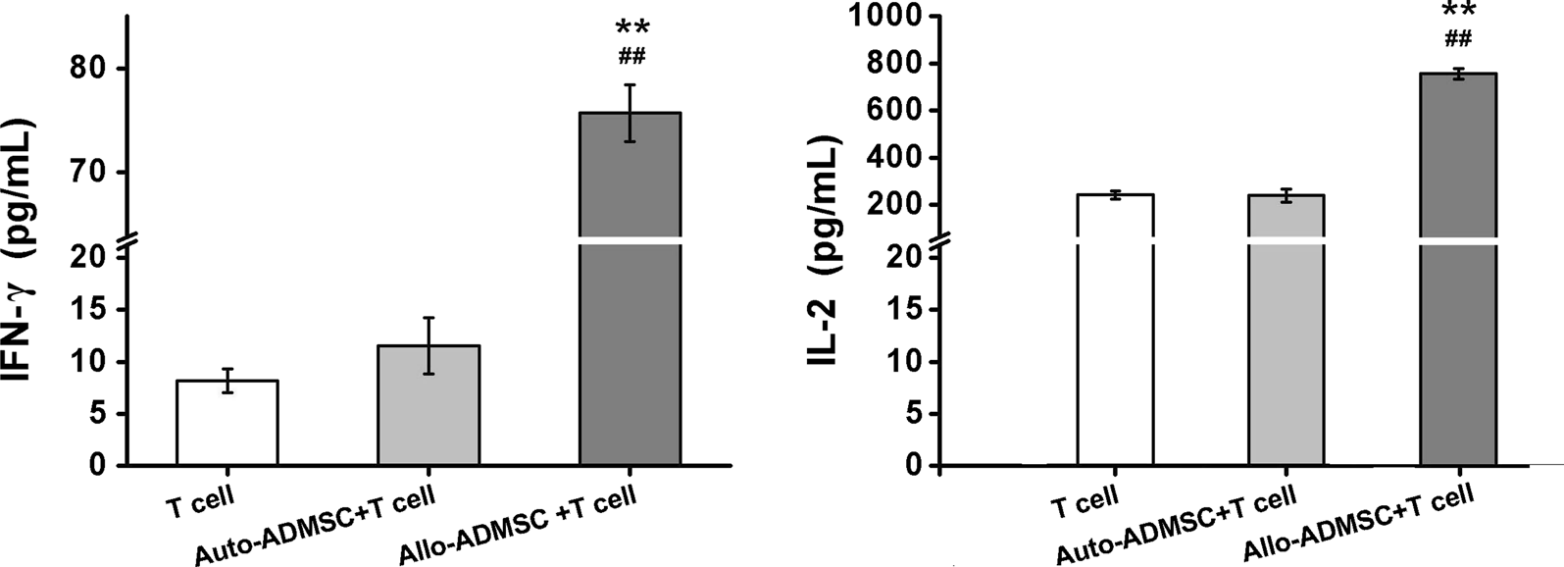
The immunogenicity of ADMSCs was analyzed using co-culture with T lymphocytes. Allo-ADMSCs added to T lymphocyte cultures increase the production of IFN-γ and IL-2 by T lymphocytes. **p < 0.01 vs. T lymphocytes, ^##^*p* < 0.01 vs. T lymphocytes plus auto-ADMSCs

To characterize the in vitro immunogenicity of ADMSCs, we evaluated the levels of IL-2 and IFN-γ in mixed T-lymphocyte cultures prepared with either allo- or auto-ADMSCs. There were no significant differences in the production of IFN-γ and IL-2 between cultures with auto-ADMSCs and cultures without ADMSCs. However, allo-ADMSCs induced a 6.56-fold increase in the production of IFN-γ and a 3.16-fold increase in the production of IL-2 when compared with auto-ADMSCs (*p* < 0.01, Fig. 4).

### Apoptosis and the survival rate of transplanted ADMSCs in ischemic brain tissues

**Fig. 5:**
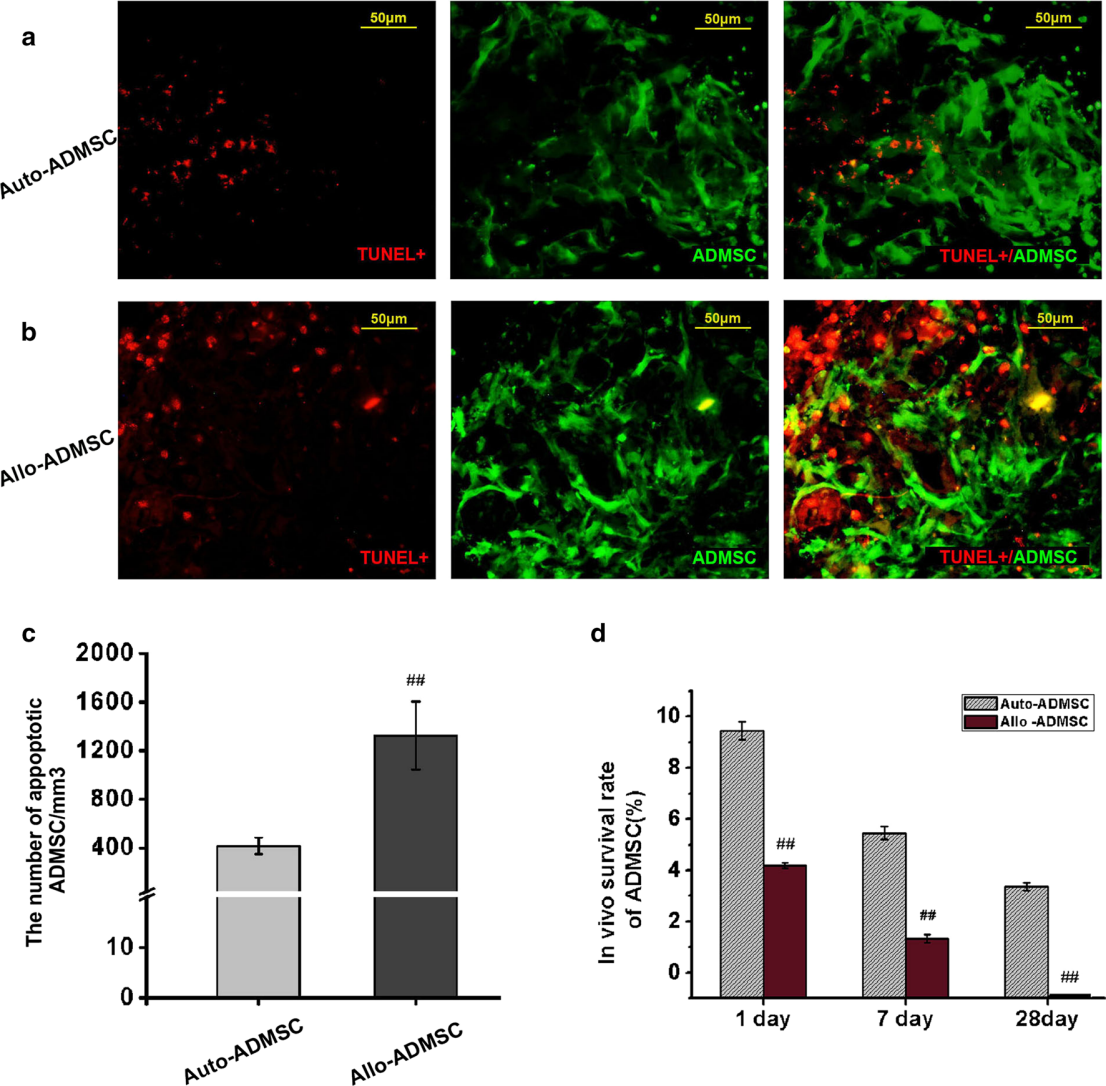
Apoptosis and survival rate of transplanted ADMSCs in ischemic brain tissues. Apoptosis of auto-ADMSCs (**a**) and allo-ADMSCs (**b**) on day 1 post transplantation was detected using a TUNEL assay. **c** Number of apoptotic transplanted ADMSCs per cubic millimeter of brain tissue near the infarct area. **d** Survival rate of transplanted auto-ADMSCs and allo-ADMSCs. TUNEL (red) and administered ADMSCs (green). Values are the mean ± SD. ^##^*p* < 0.01 vs. auto-ADMSC transplantation group

TUNEL staining was performed 1 day after transplantation to detect the apoptosis of ADMSCs. ADMSCs were tracked by transfection with EGFP (green fluorescence), and the nuclei of the apoptotic cells were labeled by red fluorescence (TUNEL+) such that EGFP- and TUNEL-positive cells represented apoptotic-transplanted ADMSCs (Fig. 5a, b). TUNEL staining showed that the number of apoptotic transplanted allo-ADMSCs per cubic millimeter of brain tissue near the infarct area was 3.19 times higher than that of apoptotic transplanted auto-ADMSCs (1323.02 ± 278.71 vs. 415.06 ± 68.79, *p* < 0.01; Fig. 5c). One day after cell transplantation, the survival rate of auto-ADMSCs was significantly higher than that of allo-ADMSCs (9.45 ± 0.34% vs. 4.19 ± 0.11%, *p* < 0.01; Fig. 5d). The survival rate of auto-ADMSCs was also significantly higher than that of allo-ADMSCs 7 days after transplantation (5.44 ± 0.25% vs. 1.33 ± 0.16%, *p* < 0.01; Fig. 5d). Twenty-eight days after cell transplantation, the survival rate of auto-ADMSCs was 3.35 ± 0.16% (Fig. 5d); however allo-ADMSCs were not observed in ischemic brain tissues at this time point.

### Migration and expression of astrocyte or neuron markers of ADMSCs in ischemic brain tissues

**Fig. 6:**
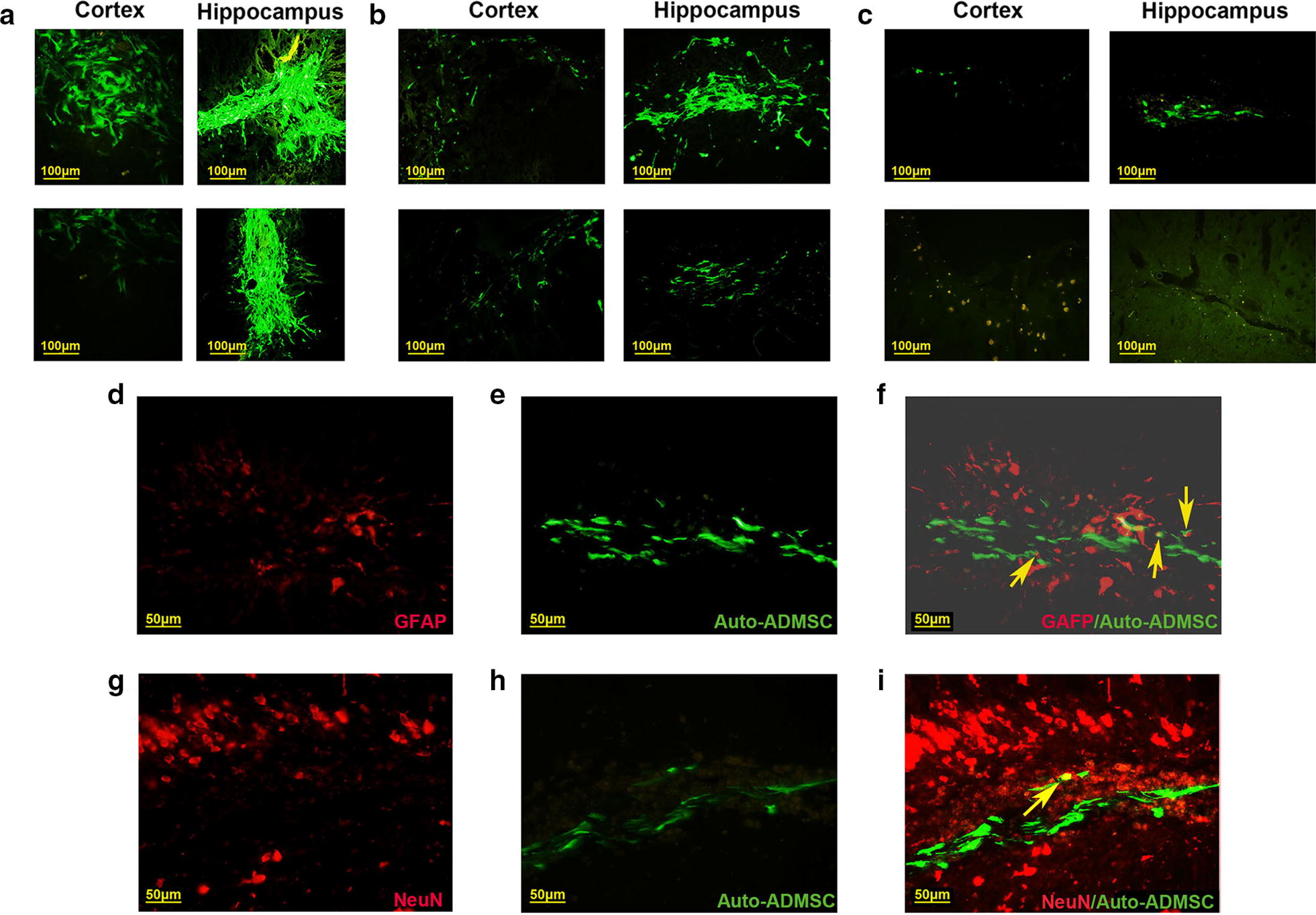
Localization and expression of astrocyte or neuron markers of transplanted ADMSCs in the injured brain tissues. Transplanted ADMSCs were detected in the cortical injury area and hippocampus 1 day (**a**), 7 days (**b**), and 28 days (**c**) after transplantation. Yellow arrows indicated that the expression of astrocyte or neuron markers in the administered auto-ADMSCs on day 28 post transplantation in the injured brain tissues. **d**–**f** Immunofluorescence microscopic images show that the administered auto-ADMSCs express astrocyte markers (GFAP) (×400). **g**–**i** The administered auto-ADMSCs express neuron markers (NeuN) (×400). NeuN (red), GFAP (red), and administered auto-ADMSCs (green)

ADMSCs labeled with EGFP were transplanted into the CA1 region of the hippocampus. One day after cell transplantation, the migration of auto-ADMSCs was observed in the penumbra cortex and hippocampus, while the migration of allo-ADMSCs was observed only in the penumbra cortex (Fig. 6a). The migration of allo-ADMSCs was observed in the hippocampus 7 days after transplantation. Overall, the migratory scope of auto-ADMSCs was wider than that of allo-ADMSCs (Fig. 6b). Twenty-eight days after cell transplantation, auto-ADMSCs were distributed mainly in the hippocampus, and to a lesser extent in the cortical injury area (Fig. 6c). However, allo-ADMSCs were not observed in either the cortical injury area or the hippocampus (Fig. 6c).

Twenty-eight days after transplantation, cells positive for EGFP (green fluorescence) and GFAP (red fluorescence) were found in the brain sections of the auto-ADMSC treatment group, indicating that the EGFP-labeled auto-ADMSCs expressed astrocyte markers. The percentage of EGFP- and GFAP-positive cells among all surviving auto-ADMSCs was 8.73 ± 0.92% (Fig. 6d–f). We also observed cells positive for EGFP and NeuN (red fluorescence) in the brain sections of the auto-ADMSC treatment group. The percentage of these immature neuron-like cells with spindle or round shapes that exhibited the expression of the apophyse marker was 2.14 ± 0.69% among all surviving auto-ADMSCs (Fig. 6g–i). Because surviving allo-ADMSCs were not observed on day 28 post transplantation, we did not examine the expression of astrocyte or neuron markers for the administered allo-ADMSCs.

### Immune response evoked by transplanted auto-and allo-ADMSCs in ischemic brain tissues

**Fig. 7:**
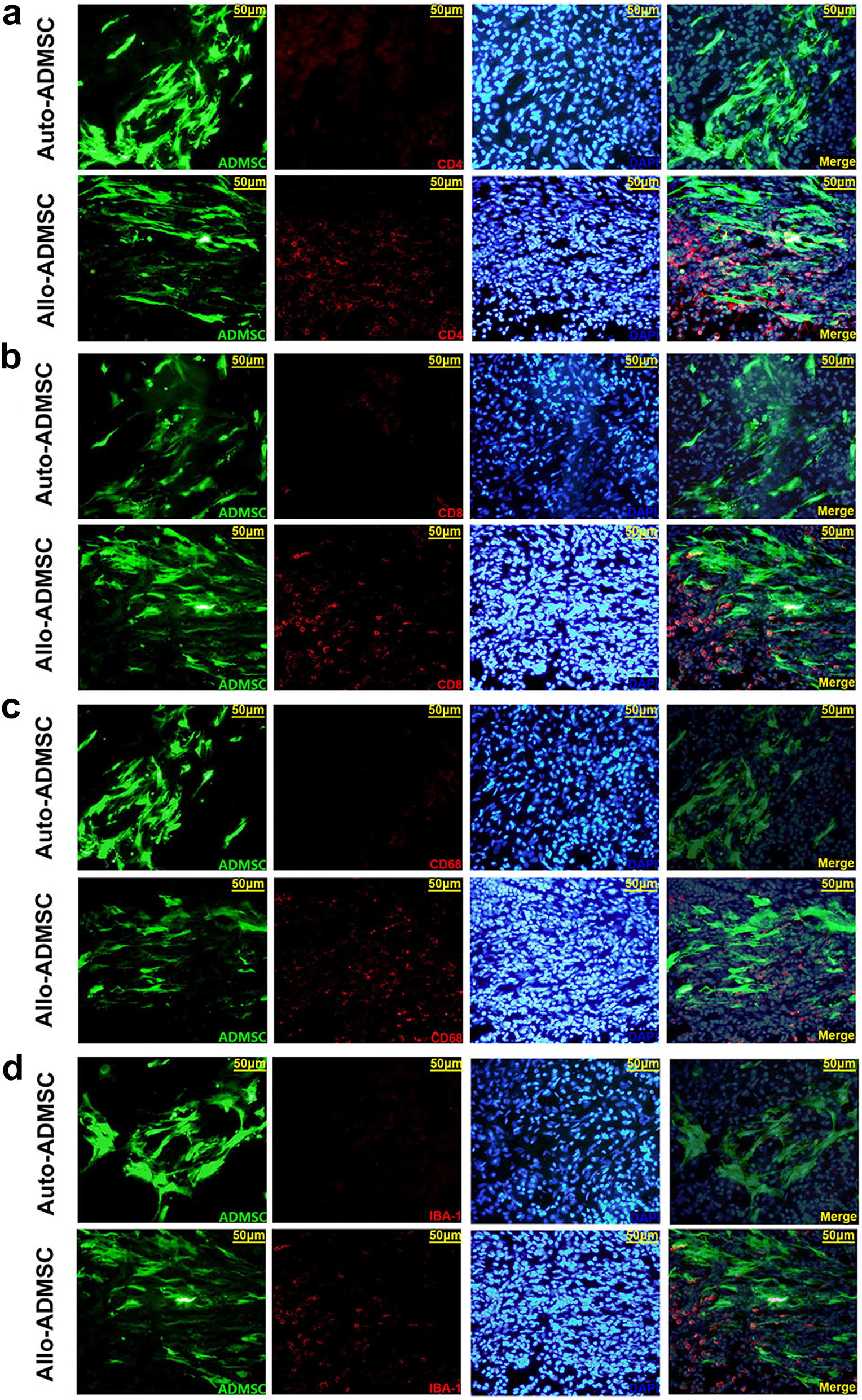
Accumulation of T lymphocytes and microglial cells in the brain tissue near the transplanted ADMSCs on day 7 post transplantation was detected by immunohistochemistry. **a** CD4+ T lymphocytes. **b** CD8+ T lymphocytes. **c** CD68+ activated microglial cells. **d** IBA-1+ resting microglial cells. Administered ADMSCs (green), CD4/CD8/IBA-1/CD68 (red), and DAPI (blue)

**Fig. 8:**
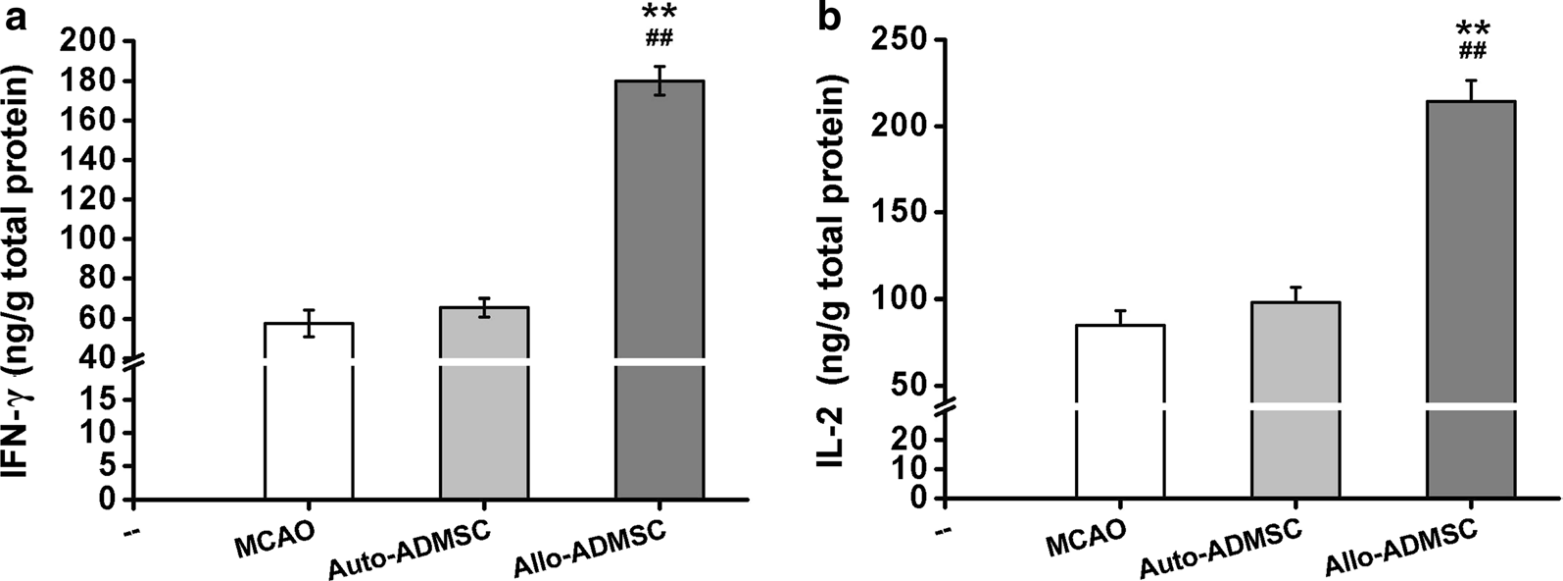
Expression of allograft rejection-related inflammatory cytokines in the brain tissue near the transplanted ADMSCs on day 7 post-transplantation was detected by ELISA. **a** IFN-γ. **b** IL-2. **p < 0.01 vs. MCAO model. ^##^*p *< 0.01 vs. auto-ADMSC transplantation group

Immunohistofluorescence analysis demonstrated that allo-ADMSCs evoked a local immunological response, characterized by an accumulation of CD4+ and CD8+ T cells, CD68+ activated microglial cells, and IBA-1+ resting microglial cells in the brain tissue near the transplanted cells at 7 days post transplantation (Fig. 7a–d), as well as a significant upregulation of the pro-inflammatory factor IFN-γ (3.12-fold, Fig. 8a) and the inflammatory cytokine IL-2 (2.53-fold, Fig. 8b) in comparison to controls (*p* < 0.01). However, these local immune responses were not observed around transplanted auto-ADMSCs.

### Auto- and allo-ADMSCs improve functional recovery after transplantation

**Fig. 9:**
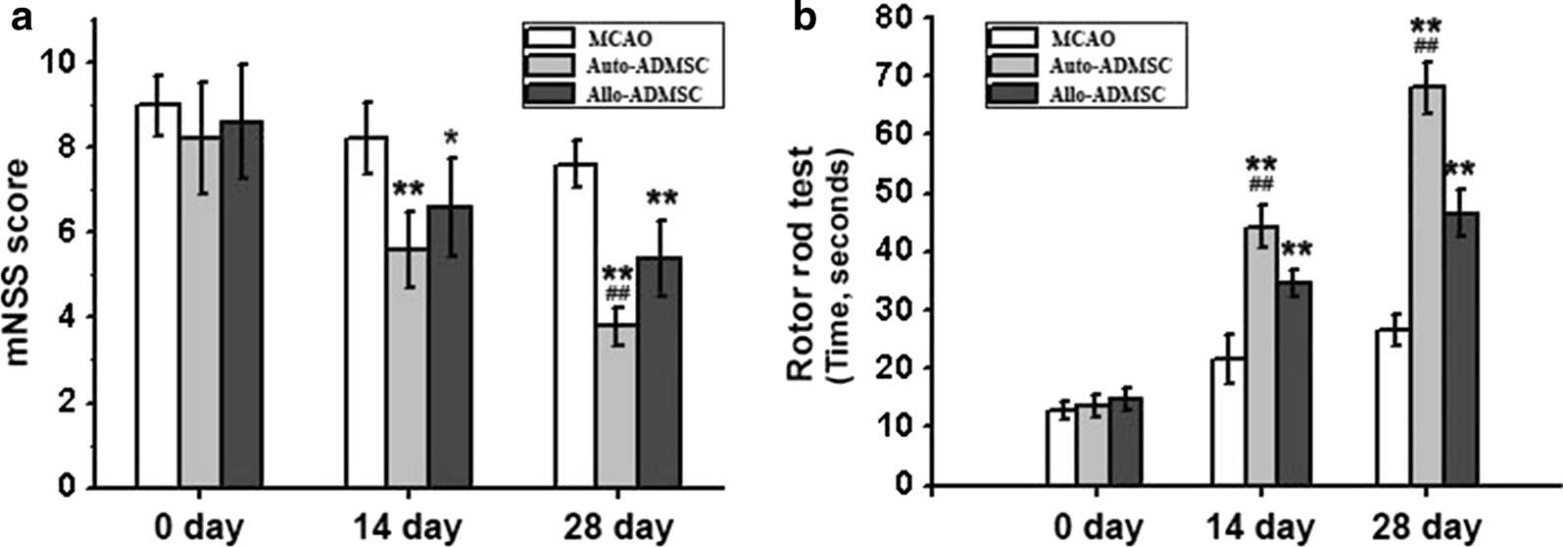
Motor function was assessed at 14 and 28 days post transplantation using the mNSS and rotarod test. Auto- and allo-ADMSCs improve functional recovery 14 and 28 days after transplantation. **a** mNSS score. **b** Rotarod test. *p < 0.05, **p < 0.01 vs. MCAO model group. ^#^*p* < 0.01 vs. auto-ADMSC transplantation group

Both auto- and allo-ADMSCs improved functional recovery after MCAO in animals at 14 and 28 days post transplantation (Fig. 9). There were no significant differences in mNSS scores between any groups prior to cell transplantation. Both transplantation groups showed significantly improved functional recovery compared with the controls, where allo-ADMSCs yielded scores of 6.6 ± 1.1 at 14 days (*p* < 0.05) and 5.4 ± 0.9 at 28 days (*p* < 0.01), and auto-ADMSCs yielded scores of 5.6 ± 0.9 at 14 days (*p* < 0.01) and 3.8 ± 0.4 at 28 days (*p* < 0.01). By comparison, the control group yielded scores of 8.2 ± 0.8 at 14 days and 7.6 ± 0.5 at 28 days. Moreover, the auto-ADMSC transplantation group showed significantly better functional recovery compared with the allo-ADMSC transplantation group 28 days after transplantation (*p* < 0.01; Fig. 9a). In the rotarod test, there were no significant differences in the mean holding time between any groups prior to cell transplantation. Allo-ADMSC (34.6 ± 2.3 s, *p* < 0.01; 46.6 ± 3.8 s, *p* < 0.01) and auto-ADMSC (44.2 ± 3.7 s, *p* < 0.01; 68 ± 4.3 s, *p* < 0.01) transplantation groups showed significantly improved functional recovery in comparison to the controls (21.6 ± 4.0 s; 26.6 ± 2.8 s) at 14 days and at 28 days, respectively. Moreover, auto-ADMSC transplantation yielded a significantly longer holding time in comparison to auto-ADMSC transplantation at 14 days (*p* < 0.01) and 28 days (*p* < 0.01) post transplantation (Fig. 9b).

### Auto- and allo-ADMSC transplantation reduces infarct size

**Fig. 10:**
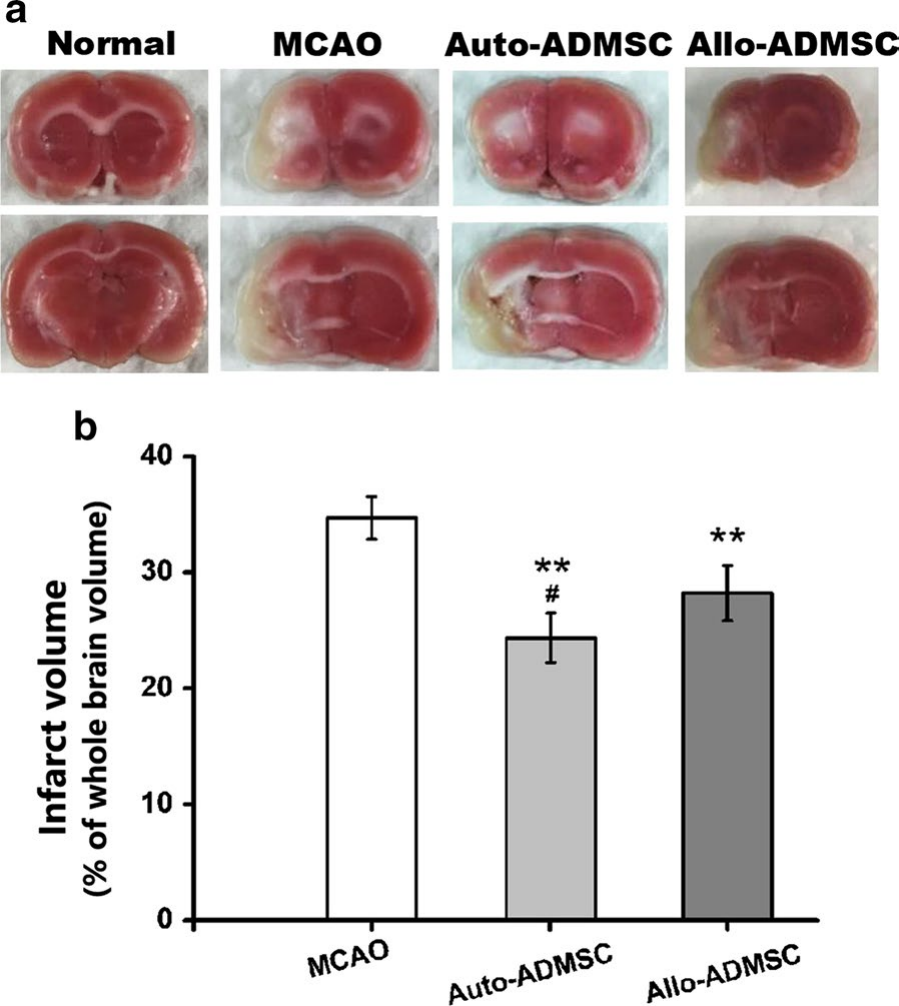
Auto-ADMSCs yielded better results in terms of reducing the infarct volume than allo-ADMSCs on day 28 post transplantation. **a** Brain sections with TTC staining (ischemic brain tissue is white). **b** Quantitative data of infarct volume from brain sections with TTC staining. **p < 0.01 vs. MCAO model group. ^#^*p *< 0.05 vs. auto-ADMSC transplantation group

Staining with TTC 28 days after transplantation (Fig. 10a) showed that both allo-ADMSC (28.22 ± 2.38%, *p* < 0.01) and auto-ADMSC transplantation (24.35 ± 2.14%, *p* < 0.01) significantly reduced relative lesion volume with respect to the MCAO model group (34.69 ± 1.84%; Fig. 10b). Moreover, auto-ADMSC transplantation reduced lesion volume to a significantly greater extent than allo-ADMSC transplantation (*p* < 0.05; Fig. 10b).

## Discussion

We investigated the immunogenicity of rat ADMSCs in vitro and compared the immunological responses to and treatment effects of the intraparenchymal administration of allo- and auto-ADMSCs after the acute phase of MCAO in rats. We found that allo-ADMSCs exhibited greater immunogenicity and evoked stronger immunological responses than auto-ADMSCs. However, auto-ADMSCs displayed a higher survival rate, longer survival time, wider migratory scope, and fewer apoptotic cells. Moreover, a small number of transplanted auto-ADMSCs expressed astrocyte-like cells and neuron-like markers 28 days after transplantation, while no surviving allo-ADMSCs were found at this time point. In addition, we found that the intraparenchymal administration of auto-ADMSCs yielded better functional recovery and reduced the infarct volume to a greater extent than allo-ADMSCs. We suggest that this effect might be associated with better viability, and migratory ability of auto-ADMSCs.

ADMSCs have been used in a number of animal models for stroke and have been demonstrated to improve functional scores and reduce infarct size [7, 11]. In recent years, ADMSCs have been identified as especially interesting for clinical applications [12, 13]. It has been reported that ADMSCs have low MHC class I marker expression and lack MHC class II markers, suggesting that they are not likely to induce rejection [9]. However, the present study shows that the expression of the MHC class I marker on ADMSCs from Lewis rats or BN rats was not low, and that MHC class II marker expression could be found on the surface of these ADMSCs. The protein expression levels of IL-2 and IFN-γ were significantly greater in the T-lymphocyte cultures with allo-ADMSCs than in those with auto-ADMSCs. These in vitro results suggest that allo-ADMSCs possess a certain level of immunogenicity and might induce rejection when transplanted to stroke model animals. The results of animal experiments in the present study have confirmed this hypothesis. When transplanted into the brains of MCAO rats, allo-ADMSCs were associated with a significant increase in the protein expression of IL-2 and IFN-γ, which are involved in inducing the inflammatory reaction and promoting allograft rejection [14]. Allo-ADMSCs were also associated with significantly increased accumulation of CD4+ and CD8+ T lymphocytes and microglial cells. CD8+ T lymphocytes can recognize MHC-I molecules, and CD4+ T lymphocytes can recognize MHC-II molecules, where the latter plays a key role in initiating immune responses. Microglial cells are the resident macrophages of the brain. The degree of activation of microglial cells represents the degree of inflammation in the brain. In addition, activated microglia secrete a variety of proinflammatory cytokines, such as interleukin-1β and tumor necrosis factor-α, which lead to further activation of microglia, resulting in a gradual inflammatory response [15]. Inhibiting the activity of microglia after cerebral ischemia can reduce the volume of cerebral infarction and protect brain tissues [16]. However, the aforementioned immune responses in the brain tissues of MCAO rats were not observed in response to auto-ADMSC transplantation.

Previous studies have shown that migration and/or implantation of xenogeneic (human) or allogeneic (rat) ADMSCs administered by intravenous infusion in the acute phase of MCAO were not detected in the injured brain at either 24 h or 14 days, but were found in various peripheral organs such as the spleen, lungs, and liver [17, 18]. The authors of these studies hypothesized that the administered xenogeneic or allogeneic ADMSCs may act indirectly on the brain by secreting several growth factors that can act to enhance endogenous repair mechanisms normally activated in the brain after a stroke. However, in the present study, the migration and implantation of intraparenchymally-administered allo- and auto-ADMSCs were observed in the brain 1 and 7 days after MCAO, while the migration and implantation of auto-ADMSCs could even be observed at 28 days after MCAO. Furthermore, we found that the migratory scope of auto-ADMSCs was wider than that of allo-ADMSCs. In addition, the survival rate of auto-ADMSCs was significantly higher than that of allo-ADMSCs on days 1, 7, and 28 after MCAO. The differences observed between the findings in the present study and previous studies could be explained by the fact that our route and timing of administration and cell sources (xenogeneic, allogeneic, or autologous) were different from those of the previous studies.

The most exciting observation in our study was that a small number of auto-ADMSCs were observed to express astrocyte or neuron markers 28 days after transplantation, while surviving transplanted allo-ADMSCs could not be found. In agreement with this finding, several previous studies have shown that rat ADSCs were able to differentiate into floating neurospheres, where in addition to being able to differentiate into neuronal- and glial-like cells, neurospheres could be induced to differentiate into Schwann cell-like cells. These Schwann cell-like cells were even able to form myelin structures with rat PC12 (pheochromocytoma cell) neurites in vitro [19, 20]. These results suggest that, aside from the growth factors secreted by surviving cells, the improved outcome owing to auto-ADMSC transplantation could have been a result of the cell replacement effect. Future studies are needed to explore the functional capacities of transplanted ADMSCs in vivo.

The majority of previous studies have focused on the effect that transplanted ADMSCs have on preserving brain tissue through the reduction of endogenous cell apoptosis [7, 21]. In the present study, we found that the number of apoptotic auto-ADMSCs was significantly lower than that of apoptotic allo-ADMSCs 1 day after transplantation, indicating that auto-ADMSCs likely elicit improved treatment outcomes.

The administration of xenogeneic or allogeneic ADMSCs has been shown to improve functional recovery independently of infarct volume in animal models of stroke [18]. Likewise, the present study demonstrated that administering auto-ADMSCs yielded better results in terms of improving functional recovery and reducing infarct volume than allo-ADMSCs. The improved effects of auto-ADMSCs are likely related to decreased immunological responses, which thereby result in higher survival rates, longer survival times, a wider migratory scope, and a lower rate of apoptosis. The differences in the observed effects on infarct volume between our study and previous studies could be explained by the fact that the route and timing of administration were different. In addition, because only two evaluations of motor function were used here, future studies are needed to compare the effects of auto- and allo-ADMSC administration on coordination, cognition, and memory impairment after stroke.

## Conclusions

Auto-ADMSCs are more effective than allo-ADMSCs in promoting recovery and reducing infarct volume in MCAO rats. This may be associated with better viability, migratory ability, and differentiation potential, and the lower degree of apoptosis in auto-ADMSCs. Future research should focus on confirming the superiority of auto-ADMSCs in the treatment of MCAO models and clarifying relevant mechanisms to provide a solid foundation for the improved clinical treatment of cerebral infarction.

## Abbreviations

ADMSCs: adipose-derived mesenchymal stem cells
Allo: allogeneic
Auto: autologous
BMSCs: bone marrow mesenchymal stem cells
EGFP: enhanced green fluorescence protein
GFAP: glial fibrillary acid protein
IFN-γ: interferon gamma
IL-2: interleukin-2
MCAO: middle cerebral artery occlusion
MHC: major histocompatibility complex
mNSS: modified neurological severity score
TTC: 2,3,5-triphenyltetrazolium chloride
